# Combined thoracoscopic and laparoscopic surgery for epiphrenic diverticulum with associated gastroesophageal reflux disease: a case report

**DOI:** 10.1186/s40792-024-01813-0

**Published:** 2024-01-15

**Authors:** Yusuke Uchi, Soji Ozawa, Tomofumi Ando, Koki Hayashi, Takuma Aoki, Motohide Shimazu

**Affiliations:** Department of Surgery, Tamakyuryo Hospital, 1401 Shimooyamada, Machida, Tokyo 194-0202 Japan

**Keywords:** Epiphrenic diverticulum, Reflux esophagitis, Thoracoscopic diverticulectomy

## Abstract

**Background:**

Surgery is indicated for symptomatic epiphrenic esophageal diverticula. Based on the features of a case, thoracoscopic or laparoscopic approaches may be used. Epiphrenic diverticula are often associated with esophageal motility disorders, but cases of reflux esophagitis have rarely been reported. In this report, we describe a case of an epiphrenic esophageal diverticulum with reflux esophagitis, which was successfully treated by thoracoscopic diverticulectomy and laparoscopic fundoplication.

**Case presentation:**

A 69-year-old man visited the hospital with a chief complaint of eructation and hiccup. Upper gastrointestinal endoscopy revealed a diverticulum in the left wall of the esophagus, which was 37–45 cm distal to the incisors. High-resolution manometry (HRM) showed no esophageal motility disorders. Due to the large size of the diverticulum, a thoracoscopic resection of the esophageal diverticulum was performed. Additionally, the patient had reflux esophagitis due to a hiatal hernia. The anti-reflux mechanism would be more impaired during the diverticulectomy; therefore, we decided that anti-reflux surgery should be performed simultaneously. Thoracoscopic esophageal diverticulectomy and laparoscopic Dor fundoplication were performed. The patient had an uncomplicated postoperative course and was discharged on the tenth operative day. He has been symptom-free without acid secretion inhibitors for 21 months after the surgery.

**Conclusions:**

We described a rare case of a large epiphrenic diverticulum with reflux esophagitis. A good surgical outcome was achieved by thoracoscopic resection of the diverticulum and laparoscopic Dor fundoplication.

## Background

Esophageal epiphrenic diverticulum is a rare disease. The estimated incidence of epiphrenic diverticula is approximately 1:500,000/year [1]. Esophageal motility disorders, such as achalasia, are often comorbidly associated with epiphrenic diverticula [2]. Surgery is generally considered to be indicated for symptomatic esophageal epiphrenic diverticula [1]. However, no unified opinions on the details of surgery and approach are currently available. In addition to resection of the esophageal diverticulum, esophageal myotomy and fundoplication may be performed depending on esophageal motility. In recent years, remarkable advances in minimally invasive surgery have been made. As a result, the choice of laparoscopy, thoracoscopy, or a combination of both in the surgical management of this disease has become controversial.

In this study, we report the successful results of thoracoscopic esophageal diverticulectomy and laparoscopic fundoplication for a case of a large epiphrenic diverticulum with diverticular erosion due to reflux esophagitis.

## Case presentation

### Chief complaints

Eructation and hiccup.

### History of present illness

A 69-year-old man was diagnosed with esophageal diverticulum 35 years ago but had been followed up without treatment. Five years ago, he visited a clinic with an exacerbation of his chief complaint. Although he was prescribed esomeprazole, his symptoms did not improve. Therefore, he was referred to our hospital for surgery.

### History of past illness

Internal hemorrhoids.

### Laboratory examinations

BUN was elevated at 25.3 mg/dL, and HBsAg was positive. No other abnormalities in blood counts or biochemical tests were reported.

### Imaging

**Fig. 1:**
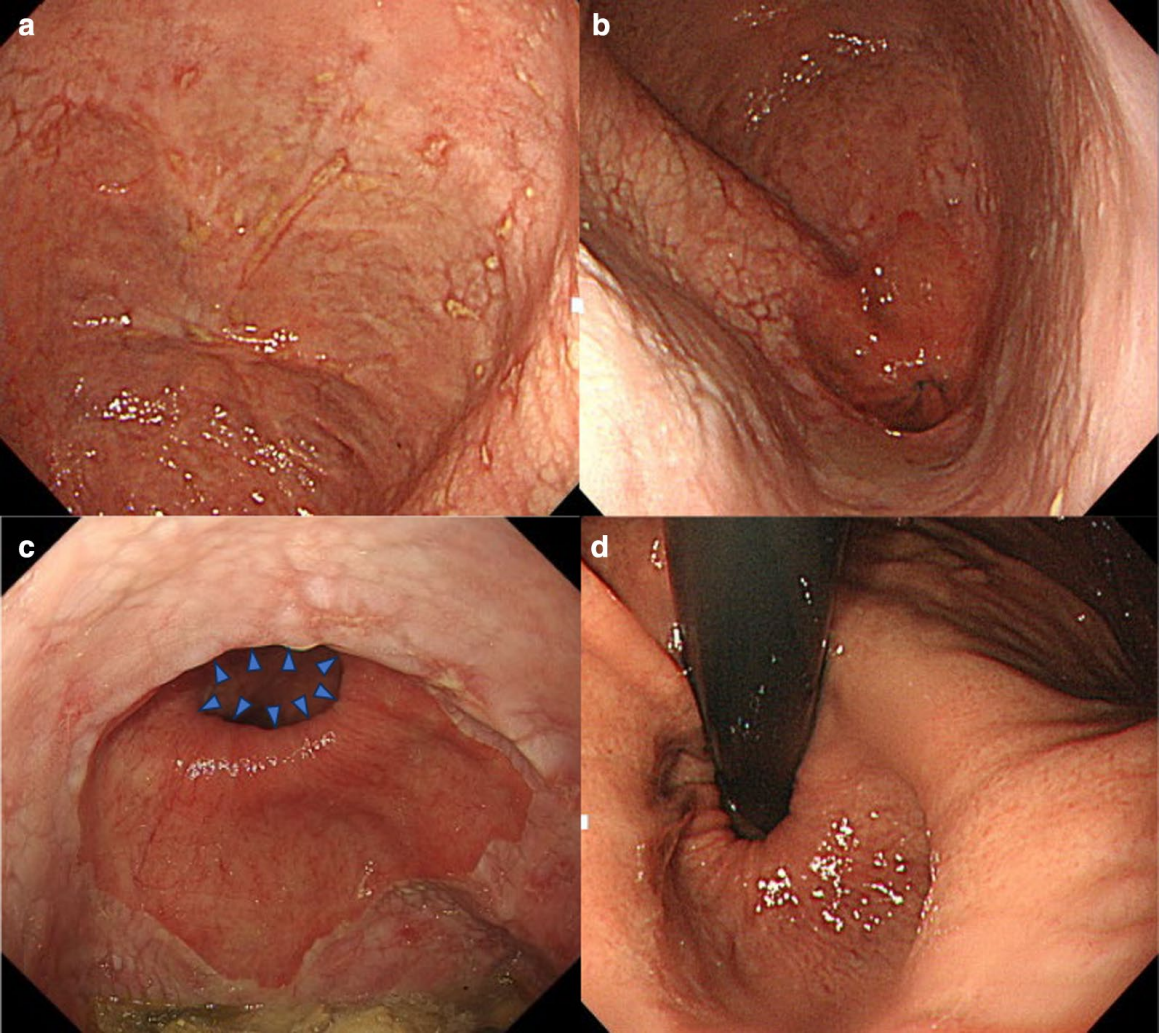
Preoperative esophagogastroduodenoscopy. **a** Multiple erosions and scars in the diverticulum. **b** The epiphrenic diverticulum is contiguous with the esophageal hiatal hernia. **c** Close view of the SCJ showing evidence of Barrett’s esophagus and esophageal erosions contiguous with the SCJ. Blue triangles showed the esophagogastric junction. **d** Esophageal hiatal hernia viewed from the stomach

**Fig. 2:**
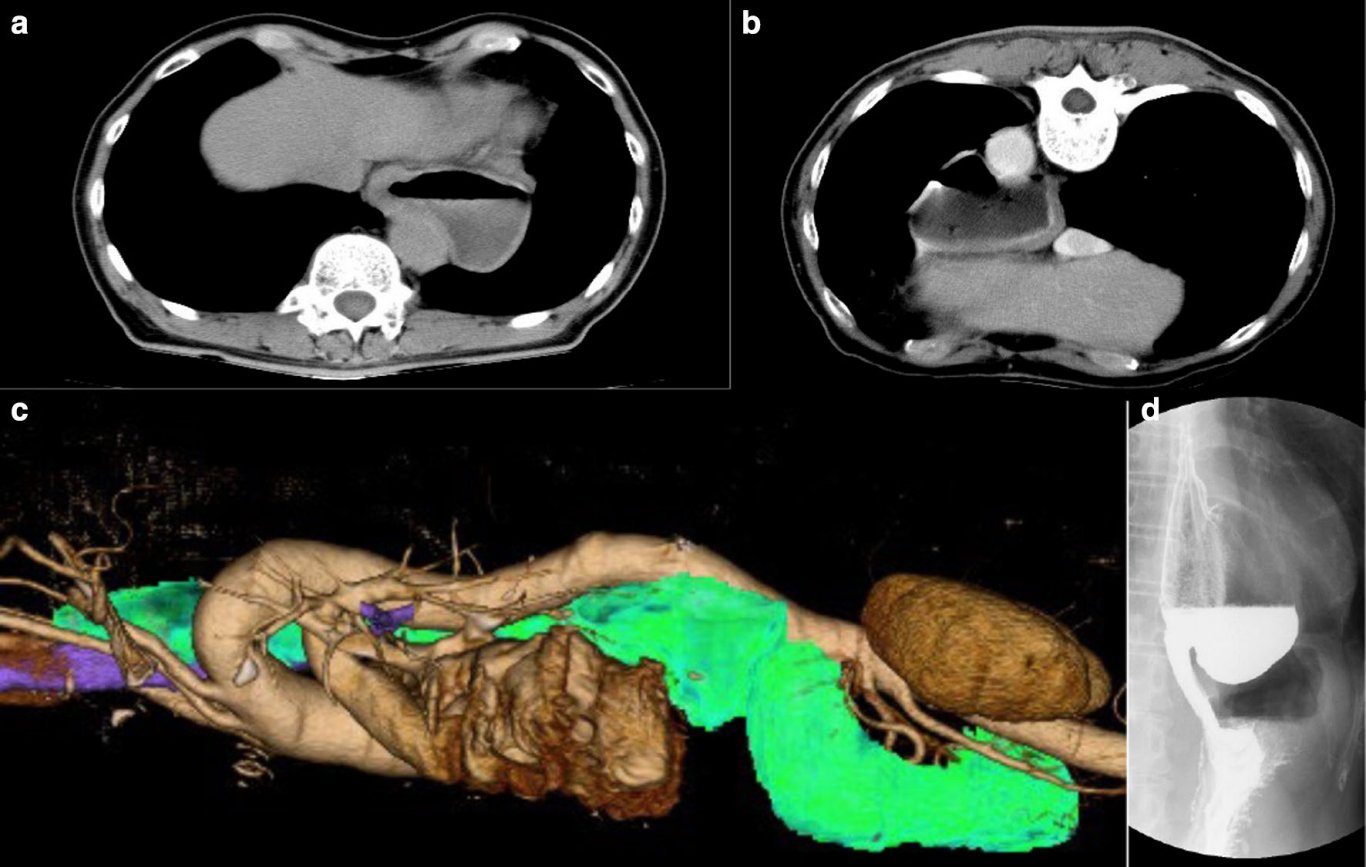
Preoperative CT and esophagogram. **a** CT in the supine position. **b** CT in the prone position. **c** 3D-CT of the epiphrenic diverticulum. **d**: Esophagogram

Upper gastrointestinal endoscopy revealed a diverticulum in the left wall of the esophagus, which was 37–45 cm distal to the incisors. After the contents of the diverticulum were drained into the stomach by changing body position, multiple erosions and scars were observed within the diverticulum. In addition, the endoscopic examination also revealed the presence of a hiatal hernia, Barrett’s esophagus, and esophageal erosions contiguous with the squamocolumnar junction (SCJ), indicative of gastric acid reflux (Fig. 1). A barium esophagogram and CT scan showed a large diverticulum with fluid accumulation on the left side of the esophagus (Fig. 2).

### High-resolution manometry

Preoperative high-resolution manometry showed no esophageal motility disorders.

### Operation

**Fig. 3:**
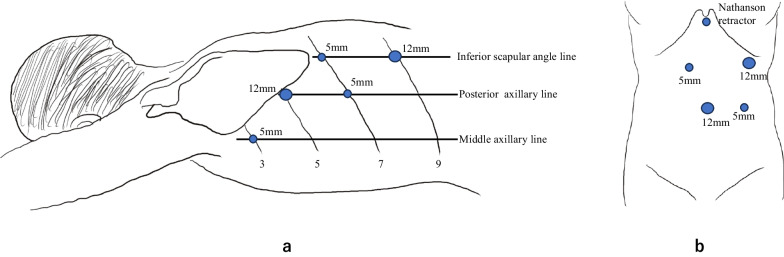
Surgical positions and trocar placement. **a** Prone position with 5 trocars during transthoracic diverticulectomy. **b** Spine position with 5 trocars and Nathanson retractor during laparoscopic Dor fundoplication

**Fig. 4:**
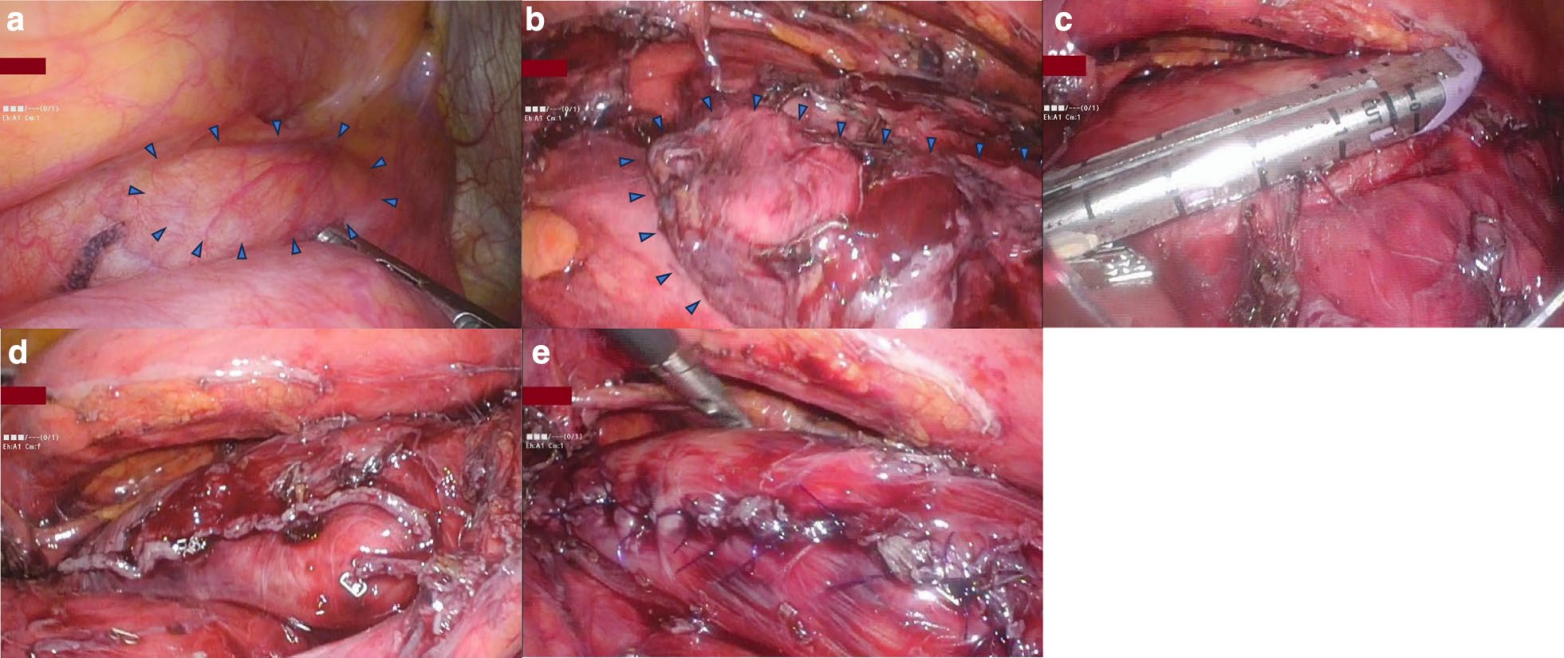
Surgical findings. Blue triangles showed the diverticulum. **a** The diverticulum before dissection. **b** The diverticulum on the left side of the esophagus after dissection. **c** Resection of the diverticulum. **d** After resection of the diverticulum. **e** After suturing the muscular layer and adventitia

The patient had a large esophageal diverticulum with reflux esophagitis and no esophageal motility disorders. Therefore, we decided to perform diverticulectomy and Dor fundoplication. The diverticulum extended 8 cm above the diaphragm. A transthoracic approach to the resection of the diverticulum was considered to be most appropriate. After induction of general anesthesia, the patient was placed in the prone position, and five trocars were inserted into the left thoracic cavity (Fig. 3). Using CO_2_ gas at 10 mmHg and 6 mmHg (after a lung collapse), a pneumothorax was induced [3]. A preoperative 3D-CT of the diverticulum was used to simulate the surgical procedure, and this simulation contributed to the success of the surgery (Fig. 2c). Thoracoscopic observation revealed that the diverticulum was located on the left side of the esophagus and was 8 cm long at the base. We inserted an endoscope into the stomach just prior to resection of the diverticulum, to prevent stenosis of the esophageal lumen during the surgery. We used the Signia™ stapling system (Covidien Japan, Tokyo) with purple staplers. This stapler can be mechanically adjusted in any direction, so that the direction of the staplers can be easily aligned with that of the diverticular resection (Fig. 4).Two 45-mm staplers and one 60-mm stapler were used to resect the diverticulum from the cranial end towards the caudal end. The overlying sutures of muscles and adventitia were added over the stapler line along its entire length using 3-0 PDSII (Fig. 4). Thereafter, the patient was placed in a supine position, and five trocars and a Nathanson retractor were inserted into the abdominal cavity (Fig. 3). The esophageal hiatus was sutured appropriately, and Dor fundoplication was performed using a laparoscopic approach.

### Postoperative course

**Fig. 5:**
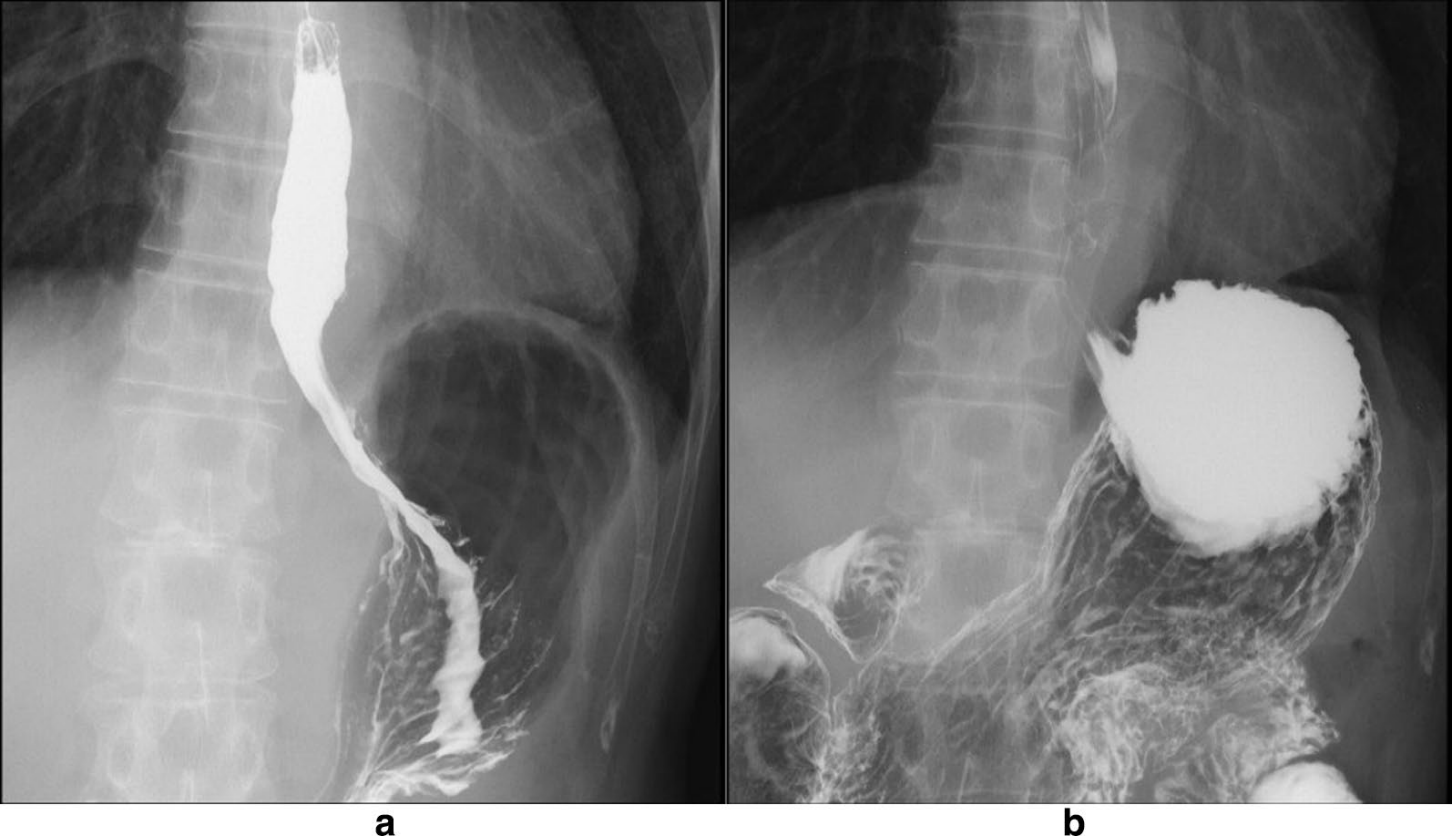
Postoperative upper gastrointestinal series. **a** Upright position. **b** Head-down position

**Fig. 6:**
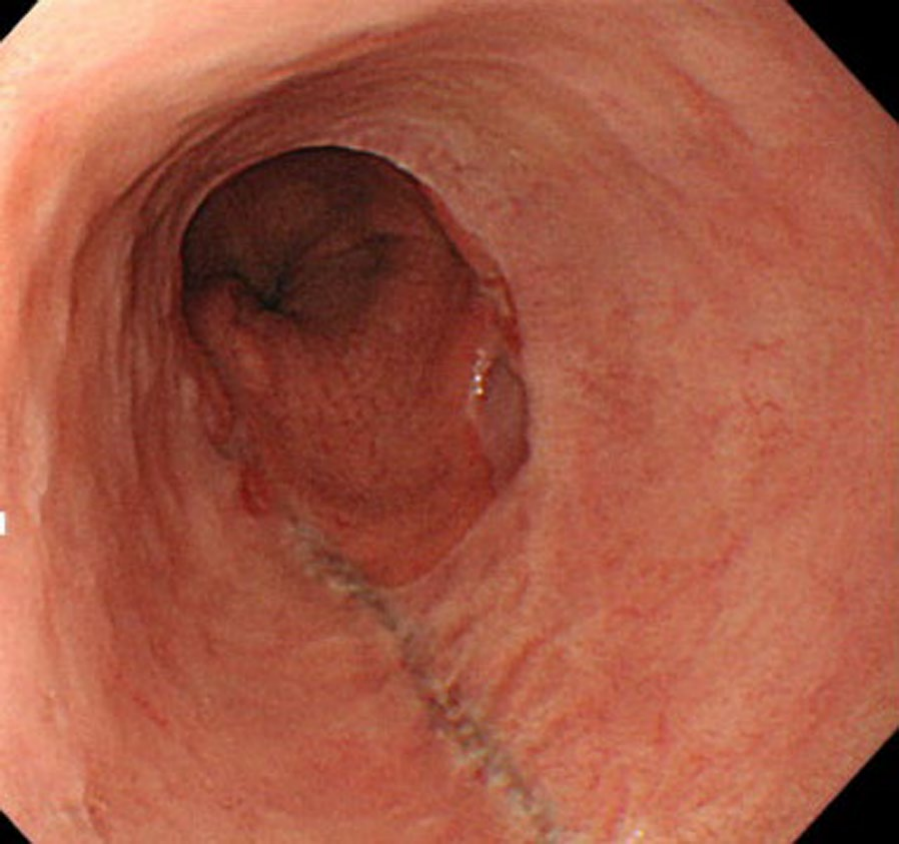
Postoperative esophagogastroduodenoscopy. No findings of reflux esophagitis or diverticular recurrence

The patient had no postoperative complications. On the second postoperative day, an esophagogram revealed that the contrast medium could flow freely into the stomach without stagnation and did not reflux into the esophagus (Fig. 5). The patient resumed oral intake on the third postoperative day and was discharged from the hospital on the tenth postoperative day. As of 21 months after surgery, the patient was not taking any acid secretion inhibitors and had no symptoms, such as reflux and dysphagia. Endoscopic findings showed no evidence of reflux esophagitis or recurrence of the diverticulum (Fig. 6).

## Discussion

We encountered a rare case of a giant epiphrenic diverticulum associated with gastroesophageal reflux disease. We performed thoracoscopic diverticulectomy and laparoscopic fundoplication for the hiatal hernia and gastroesophageal reflux disease, while avoiding esophagomyotomy due to absence of any evidence of underlying esophageal motility disorders, and obtained excellent surgical results.

Epiphrenic esophageal diverticulum is a rare condition, and the indication for surgery depends on symptoms. Conservative management and radiologic or endoscopic follow-up are acceptable in cases of asymptomatic or minimally symptomatic epiphrenic diverticulum. Surgery is generally indicated in cases of severe dysphagia, regurgitation, or retention of contrast material during esophagography, and the risk of aspiration pneumonia is considered high [4]. On the other hand, some clinicians suggest that surgery should be performed for all epiphrenic diverticula, regardless of the presence or absence of symptoms [5]. In this case, the patient visited our hospital with worsening symptoms of eructation. Since a large esophageal diverticulum was thought to cause the symptoms, we decided that surgery was indicated. After surgery, the patient’s symptoms improved, and the absence of signs of impaired transit and reflux on the esophagogram resulted in a favorable outcome.

Small esophageal diverticula tend to shrink with myotomy only, and resection may be unnecessary [6, 10]. On the other hand, large diverticula require resection [1, 6]. Traditionally, diverticulectomy is performed by open thoracotomy (especially left open thoracotomy), which is associated with a high complication rate and postoperative mortality [8, 9, 11]. In recent years, however, with the development of minimally invasive surgery, use of thoracoscopy, laparoscopy, or both procedures have been widely reported. Minimally invasive surgery has been reported to have a lower perioperative mortality rate than open surgery [12]. Laparoscopic surgery allows adequate observation of the esophagogastric junction when performing a myotomy or a fundoplication. The advent of the endo stapler, which allows resection of the diverticular neck parallel to the esophageal axis, has popularized laparoscopic surgery. However, the distance between the hiatal hernia and diverticulum, size of the diverticulum, and adhesions between the diverticular wall and pleura limit the sole use of laparoscopy for diverticulectomy [1, 12–15]. Thoracoscopic surgery, conversely, provides excellent visualization and maneuverability for the dissection of large diverticula and diverticula with surrounding areas of inflammation [16]. Some authors have suggested that a transthoracic resection (open or thoracoscopic) should be followed by a laparoscopic myotomy and fundoplication for large diverticula [1]. According to Achim et al., diverticula should be approached by thoracoscopy for cases where the diverticular oral end is > 5 cm away from the esophagogastric junction and by laparoscopy only for patients with diverticula that are < 5 cm away [6]. However, few reports of combined laparoscopic and thoracoscopic surgery have been made. Brandeis et al. reported a case series of minimally invasive surgery for epiphrenic diverticula. Among the 189 patients who underwent minimally invasive surgery, only 12 (6.3%) patients underwent the combined laparoscopy and thoracoscopy [17]. In this case, the patient had a large diverticulum, which had a length of 8 cm and posed a challenge for resection solely by laparoscopy. The thoracoscopic approach facilitated a safe and reliable resection of the diverticulum.

After diverticulectomy, suture failure from the staple line is the most severe complication. Addition of adventitia and muscular layer sutures along the staple line has been reported to decrease suture failure rate. In clinical practice, a number of clinicians routinely use this approach [16]. In this case, endo staplers were used for diverticulectomy. Thereafter, adventitia and muscular layer sutures were added along the entire staple line length without evidence of postoperative suture failure.

Epiphrenic diverticula are often associated with esophageal motility disorders [1]. Therefore, some clinicians suggest that esophagomyotomy should be performed as a primary procedure. A review of several previous reports shows that suture failure and recurrence rates are higher when esophageal myotomy is not performed [1, 6]. On the other hand, several clinicians have recommended the use of elective myotomy [4, 7–9]. The significance of myotomy is to decrease intraesophageal pressure, and its use in patients without esophageal motility disorders or increased LES pressure might be questionable. Additionally, it may promote postoperative reflux esophagitis [4]. In this case, high-resolution manometry showed no esophageal motility disorders; therefore, myotomy was not performed. As a result, the patient had a good outcome with no postoperative complications, such as suture failure.

Reflux esophagitis is rarely associated with epiphrenic diverticulum, which often develops in association with esophageal motility disorders. A search of the PubMed database did not reveal any case reports of epiphrenic diverticulum with reflux esophagitis. In this case, preoperative upper gastrointestinal endoscopy revealed multiple erosions and scars within the diverticulum. Upper gastrointestinal endoscopy also revealed a hiatal hernia, Barrett’s esophagus, and esophageal erosions contiguous with the SCJ, indicative of the presence of gastric acid reflux. Therefore, the erosions and scars observed in the diverticulum were thought to be associated with the reflux esophagitis. The anti-reflux mechanism was considered more impaired during the diverticulectomy procedure; therefore, we decided that anti-reflux surgery was desirable and performed laparoscopic Dor fundoplication. Partial fundoplication was chosen to prevent the risk of postoperative dysphagia. The patient reported no postoperative symptoms, suggesting that the appropriate procedures were performed.

## Conclusions

In this report, we described a rare case of a large epiphrenic diverticulum with associated reflux esophagitis. A good surgical outcome was achieved by thoracoscopic resection of the diverticulum and laparoscopic Dor fundoplication.

## Data Availability

The data used in this study are available from the corresponding author upon reasonable request.

## Abbreviations

HRM: High-resolution manometry
SCJ: Squamocolumnar junction
LES: Lower esophageal sphincter
